# Time series analysis of neoadjuvant chemotherapy and bevacizumab-treated breast carcinomas reveals a systemic shift in genomic aberrations

**DOI:** 10.1186/s13073-018-0601-y

**Published:** 2018-11-29

**Authors:** Elen Kristine Höglander, Silje Nord, David C. Wedge, Ole Christian Lingjærde, Laxmi Silwal-Pandit, Hedda vdL Gythfeldt, Hans Kristian Moen Vollan, Thomas Fleischer, Marit Krohn, Ellen Schlitchting, Elin Borgen, Øystein Garred, Marit M. Holmen, Erik Wist, Bjørn Naume, Peter Van Loo, Anne-Lise Børresen-Dale, Olav Engebraaten, Vessela Kristensen

**Affiliations:** 1 Department of Genetics, Institute for Cancer Research, Oslo University Hospital Radiumhospitalet, Postboks 4953 Nydalen, 0424 Oslo, Norway; 2 KG Jebsen Center for Breast Cancer Research, Institute for Clinical Medicine, University of Oslo, Oslo, Norway; 3 Big Data Institute, University of Oxford, Oxford, UK; 4 Biomedical Informatics, Department of Informatics and Centre for Cancer Biomedicine, University of Oslo, Oslo, Norway; 5 Department of Oncology, Oslo University Hospital, 0407 Oslo, Norway; 6 Section for Breast and Endocrine Surgery, Oslo University Hospital, Oslo, Norway; 7 Department of Pathology, Oslo University Hospital, Oslo, Norway; 8 Department of Radiology, Oslo University Hospital, Oslo, Norway; 9 Cancer Research UK London Research Institute, London, UK; 10 Department of Clinical Molecular Biology (EpiGen), Divison of Medicine, Akershus University Hospital, Lørenskog, Norway; 11 Institute of Clinical Medicine, University of Oslo, Oslo, Norway

**Keywords:** Breast cancer, Tumor heterogeneity, Clonal and subclonal aberrations, Chemotherapy, Targeted treatment, Angiogenesis

## Abstract

**Background:**

Chemotherapeutic agents such as anthracyclines and taxanes are commonly used in the neoadjuvant setting. Bevacizumab is an antibody which binds to vascular endothelial growth factor A (VEGFA) and inhibits its receptor interaction, thus obstructing the formation of new blood vessels.

**Methods:**

A phase II randomized clinical trial of 123 patients with Her2-negative breast cancer was conducted, with patients treated with neoadjuvant chemotherapy (fluorouracil (5FU)/epirubicin/cyclophosphamide (FEC) and taxane), with or without bevacizumab. Serial biopsies were obtained at time of diagnosis, after 12 weeks of treatment with FEC ± bevacizumab, and after 25 weeks of treatment with taxane ± bevacizumab. A time course study was designed to investigate the genomic landscape at the three time points when tumor DNA alterations, tumor percentage, genomic instability, and tumor clonality were assessed. Substantial differences were observed with some tumors changing mainly between diagnosis and at 12 weeks, others between 12 and 25 weeks, and still others changing in both time periods.

**Results:**

In both treatment arms, good responders (GR) and non-responders (NR) displayed significant difference in genomic instability index (GII) at time of diagnosis. In the combination arm, copy number alterations at 25 loci at the time of diagnosis were significantly different between the GR and NR. An inverse aberration pattern was also observed between the two extreme response groups at 6p22-p12 for patients in the combination arm. Signs of subclonal reduction were observed, with some aberrations disappearing and others being retained during treatment. Increase in subclonal amplification was observed at 6p21.1, a locus which contains the VEGFA gene for the protein which are targeted by the study drug bevacizumab. Of the 13 pre-treatment samples that had a gain at VEGFA, 12 were responders. Significant decrease of frequency of subclones carrying gains at 17q21.32-q22 was observed at 12 weeks, with the peak occurring at TMEM100, an ALK1 receptor signaling-dependent gene essential for vasculogenesis. This implies that cells bearing amplifications of VEGFA and TMEM100 are particularly sensitive to this treatment regime.

**Conclusions:**

Taken together, these results suggest that heterogeneity and subclonal architecture influence the response to targeted treatment in combination with chemotherapy, with possible implications for clinical decision-making and monitoring of treatment efficacy.

**Trial registration:**

NCT00773695. Registered 15 October 2008

**Electronic supplementary material:**

The online version of this article (10.1186/s13073-018-0601-y) contains supplementary material, which is available to authorized users.

## Background

Breast cancers encompass a heterogeneous group of tumors. While most breast carcinomas are estrogen receptor positive, and hence eligible for hormone therapy, a large proportion of these patients also receive chemotherapy, which significantly improves the outcome. Chemotherapeutic agents such as anthracyclines and taxanes are commonly used in the neoadjuvant setting for reduction of tumor size prior to surgery. Obtaining pathological complete response (pCR) after neoadjuvant treatment is an indicator of better prognosis [1–3]. Highly proliferative tumors have been shown to respond best to treatment with both anthracyclines and taxanes [4], but only a fraction (10–20%) of those receiving neoadjuvant treatment will reach pCR, which emphasizes the need for improved predictive markers [1, 5, 6], an area of focused research [7–10]. One approach to improve response rates is to introduce combinations of targeted therapies. For example, bevacizumab is an antibody which binds to vascular endothelial growth factor A (VEGFA) and inhibits its receptor interaction, thus obstructing the formation of new blood vessels. The VEGF receptor (VEGFR) has in addition many downstream effectors that can lead to multiple tumor-associated phenotypes such as increased proliferation, cell motility, angiogenesis, and vascular permeability [11–14]. Therefore, blocking VEGFA could affect all of these functions. Larger studies have reported an increased rate of pCR in patients treated with chemotherapy in combination with bevacizumab, but the phenotypic characteristics linked to tumor response by antiangiogenic therapy are largely unknown [15–18].

Tumor heterogeneity results from different phenotypic profiles of the tumor cells, including cellular morphology, proliferation, and metastatic potential, and may strongly influence treatment response. Tumor cells continuously accumulate genomic changes, some of which can lead to the selection and growth advantage of certain cells, leading to subsequent clonal expansion [19]. Several lineages may develop in parallel and result in the observed heterogeneity [20]. Eliminating a specific subclone may not be sufficient to observe tumor shrinkage; instead, attacks must be directed against multiple clones and therapy may need to be changed as the dynamics of the tumor cell subpopulations can shift during treatment. Copy number alterations (CNAs) may be useful markers to follow tumor clonality in heterogeneous tumors. We have previously developed tools for the estimation of allele-specific CNAs, as well as the clonal composition of tumors (the ASCAT and Battenberg algorithm, respectively) [20, 21]. In this study, ASCAT and Battenberg analyses were employed to study the time course of genomic instability through the analysis of CNAs in Her2-negative breast cancer patients treated in a neoadjuvant setting with chemotherapy with or without bevacizumab. The goal of this study was to identify CNAs, clonal and subclonal, that may lead to the identification of markers predicting treatment response. A time course study was designed to investigate the dynamics of copy number aberrations in tumor DNA during treatment of breast cancer patients.

## Methods

### Patient material and study design

Material was collected from women with breast cancer included in a randomized phase II trial evaluating the efficacy and safety of bevacizumab (Avastin; Genentech, Inc., South San Francisco, CA) in combination with neoadjuvant treatment regimens. The inclusion criteria were met by patients with large (> 2.5 cm) Her2-negative tumors, with no signs of metastatic disease, and without receiving prior treatment. Written informed consent forms were obtained from all patients. The study was approved by the Institutional Protocol Review Board of Oslo University Hospital, the Regional Committee for Medical and Health Research Ethics for South-Eastern Norway, and the Norwegian Medicines Agency and was carried out in accordance with the Declaration of Helsinki, International Conference on Harmony/Good Clinical practice. The study was registered in the https://clinicaltrials.gov/ database with the identifier NCT00773695.

Women in both treatment arms of the study received four cycles of FEC100 (fluorouracil (5FU) 600 mg/m^2^, epirubicine 100 mg/m^2^, and cyclophosphamide 600 mg/m^2^) given every 3 weeks, followed by 12 weekly infusions with paclitaxel (80 mg/m^2^) or four cycles with docetaxel (100 mg/m^2^), one every 3 weeks. Patients randomly assigned to the bevacizumab treatment arm received a 15-mg/kg dose on day 1 of each chemotherapy course, or a 10-mg/kg dose every other week when receiving paclitaxel. In a separate cohort of the study, a small subset of patients above 55 years of age and with hormone receptor-positive tumors were treated with aromatase inhibitors (*n* = 12). The randomization to bevacizumab in this subcohort was independent from the cohort receiving chemotherapy. In the presented study, only the patients who received chemotherapy with or without bevacizumab were included in the analyses.

Tumor tissue was obtained by ultrasound-guided 14- or 16-gauge needle biopsy prior to treatment and after 12 weeks of treatment. The third biopsy was taken at the time of surgery (week 25). Surgery was performed 4 weeks after administration of the last dose of bevacizumab. Biopsies from all three time points were frozen in liquid nitrogen and stored at − 70 **°**C. If possible, peripheral blood was also obtained at all three time points.

Patients were classified as having pathological complete response (pCR) or non-pCR. Pathological complete response was obtained if there were no tumor cells detected in the surgery specimen, nor lymph nodes. Shrinkage of the tumor was measured, and a continuous response ratio was calculated as the tumor size at surgery divided by tumor size at diagnosis. The tumor size prior to treatment and at 12 weeks was measured by MRI, ultrasound, or mammography. MRI measurements were used for tumor classification and for the evaluations performed in the majority of patients. In 22 patients, MRI was not available, and the largest measured diameter of either ultrasound or mammography was used. The patients were divided into three groups based on percent shrinkage of the tumor from diagnosis to surgery: good response (GR) (*n* = 33, more than 90% shrinkage, RR 0–0.0976), intermediate response (IR) (*n* = 68, between 10 and 90% shrinkage, RR 0.106–0.83), and no response (NR) (*n* = 22, less than 10% shrinkage, RR 0.903–2.35).

Patients treated only with chemotherapy were classified as belonging to the chemotherapy arm, while patients treated with combination of chemotherapy and bevacizumab were classified within the combination arm.

### DNA and RNA extraction

Fresh frozen tumor biopsies were dissected into small pieces, mixed, and divided into amounts suitable for DNA, RNA, and protein extraction. DNA was isolated using the QIAcube and AllPrep DNA/RNA Mini Kit 350 or 600 for biopsies from the first two or the last time point, respectively (Qiagen). The company’s standard protocol was followed.

Total RNA was extracted using TRIzol® reagent (Thermo Fisher Scientific), according to the manufacturer’s instructions. RNA concentration was measured using the NanoDrop® ND-1000 Spectrophotometer (Thermo Fisher Scientific), and RNA integrity assessed using the 2100 Bioanalyzer (Agilent Technologies).

### mRNA expression and proliferation score

Expression profiling was performed using 40 ng total RNA from each tumor biopsy analyzed by one color Sureprint G3 Human GE 8 × 60 k Microarrays (Agilent Technologies), according to the manufacturer’s protocol. The arrays were scanned using a Microarray Scanner with Sure Scan High Resolution Technology (Agilent Technologies), and the raw microarray images were processed using Feature Extraction software (v10.7.3.1; Agilent Technologies). The data was quantile normalized applying Bioconductor package limma, and missing values were imputed using Bioconductor package pcaMethods.

The proliferation score was calculated as the mean normalized expression of the 11 proliferation genes included in PAM50 [22]: *CCNB1*, *UBE2C*, *BIRC5*, *NDC80*, *CDC20*, *PTTG1*, *RRM2*, *MKI67*, *TYMS*, *CEP55*, and *NUF*.

### Copy number analysis of tumors

Tumor DNA was analyzed for CNAs using Genome-Wide Human SNP array 6.0 (Affymetrix). For tumors where clinical response data was available, copy number profiles were calculated from 123 biopsies from the time of diagnosis and 111 and 110 biopsies after 12 and 25 weeks of treatment, respectively. Raw data was normalized to HapMap using Affymetrix Power tools. The resulting copy number profiles were segmented with the allele-specific piecewise constant fitting (ASPCF) algorithm [23], and subsequently, the allele-specific copy number analysis of tumors (ASCAT) tool [21] was used to estimate tumor cell fraction, tumor ploidy, and copy number. When available, matched blood was used as a reference; otherwise, germline genotypes were predicted using the built-in function *predictGG* in ASCAT. ASCAT is dependent on a sufficient amount of the sample DNA bearing CNAs to accurately estimate aberrant tumor cell fraction. Otherwise, tumors are classified as “non-aberrant.” The tumor cell fraction of the non-aberrant samples was manually assessed, based on the copy number profile and additional tumor percent estimates from the pathologist. If the copy number profile was flat and the pathologist estimated 0% tumor cells, the tumor cell fraction was set to zero. If the tumor had non-aberrant copy number profile at week 0 or week 12, but not the other time points, the tumor cell percentage at that time point was considered unknown. Clonal and subclonal events were estimated with the Battenberg algorithm [20]. The genomic instability index (GII) was measured as the fraction of aberrant probes throughout the genome above or below ploidy. Student’s *t* test was applied to test difference in mean GII between patients with pCR versus non-pCR. Analysis of variance (ANOVA) was applied when testing differences in mean GII between the three response groups: GR, IR, and NR. Pearson correlation was applied to assess the strength of the relationship between GII and proliferation score.

For each sample, an aberration score was calculated per segment. Total copy number per segment was classified as a gain if it was greater than (ploidy + 0.6) or a deletion if it was less than (ploidy − 0.6). Gains and amplifications were analyzed as one event. Remaining segments were scored as non-aberrant. Frequency plots were generated based on the aberration score across all samples per segment.

LogR estimates adjusted for tumor cell fraction and ploidy were calculated based on the ASCAT output and equations. The total copy number, adjusted for tumor percent, was divided by the sample’s calculated ploidy and subsequently log2-transformed and multiplied with the array-noise-factor, *γ* (*γ* = 0.55). The logR estimates were filtered based on the correlation to mRNA expression (Pearson correlation ≥ 0.5), thereby also removing most CNVs as they have documented limited on gene expression [24]. Subsequently, Student’s *t* test was performed to study the difference in mean logR between the two extreme response groups GR and NR. Multiple testing correction was performed by the Benjamini-Hochberg method.

### Clonal and subclonal tumor composition analysis

In order to identify changes in tumor composition during treatment, first, a reference sample was picked. This was usually the sample from the week 0. However, for four patients, the week 0 sample had very low cellularity and better profiles were obtained from week 12, and hence, this was used as reference samples for these four patients. Fifteen samples could not be further analyzed as neither week 0 nor week 12 time point yielded satisfactory Battenberg profiles. The aberrant cell fraction (ACF) of the reference sample was estimated by the Battenberg output as described in [20]. The ACFs of the later time points were estimated using either Battenberg estimates, for samples with good Battenberg profiles, or the position of the main peak in the density plot of ACFs calculated for each reference segment. Samples that are diploid in the reference sample (ploidy < 3) were used to identify segments that have just one aberrant copy number state, i.e., segments that are clonal and aberrant or that are subclonal and where one of the states are non-aberrant. Based on this, aberrant segments were categorized as clonal or subclonal and as either loss, gain, or LOH. For each segment, the fraction of cells bearing the CNA was estimated for each time point, assuming that the aberrant state per cell was the same at all time points. The total number of samples that showed an increase or a decrease in clonality with time during treatment in each segment was calculated. Increase/decrease in subclonality is determined separately in each 12- or 25-week sample, relative to the diagnosis sample. The number of increases/decreases is then summed across all patients. We expect segments that have no selective pressure to have the same number of increases and decreases, on average, across all tumors. A chi-squared test followed by Benjamini-Hochberg multiple testing correction was used to test whether there were significantly more increases than decreases (or vice versa) in clonality in each segment. Segments under positive selection will have more tumors with an increase in clonality than a decrease. Segments under negative selection will have more samples showing a decrease in clonality than an increase.

## Results

Patient biopsies were taken at diagnosis (week 0) and during treatment (weeks 12 and 25), from patients included in the study, randomly assigned to a treatment arm. At the time of diagnosis, quality copy number profiles were captured from 123 patient biopsies. Twenty-three patients achieved pCR, and 100 patients were characterized with non-pCR. A pCR was reported twice as often in the combination arm as in the chemotherapy arm (15 versus 8). The clinical parameters and results are thoroughly described by Silwal-Pandit et al. [25]. Almost equal numbers of non-pCR patients were observed in both treatment arms. Twelve of the patients with pCR had estrogen receptor (ER)-positive tumors, and nine were ER negative.

The ratio between tumor size at time of surgery and time of diagnosis (response ratio, RR) was calculated and ranged from 0 to 2.35. Patients were categorized as having good, intermediate, or no response (GR, IR, NR) as described in the “Methods” section. Despite the significant reduction of tumor mass, 11 patients in the GR group were not classified as having pCR, due to positive node status and/or a few tumor cells observed by the pathologist at week 25. The association of these treatment groups to clinical and molecular parameters such as ER status, molecular subtype, and clinical presentation are described elsewhere [25].

### CNAs in relation to tumor characteristics such as genomic instability and proliferation index for responders and non-responders in both treatment arms

**Fig. 1:**
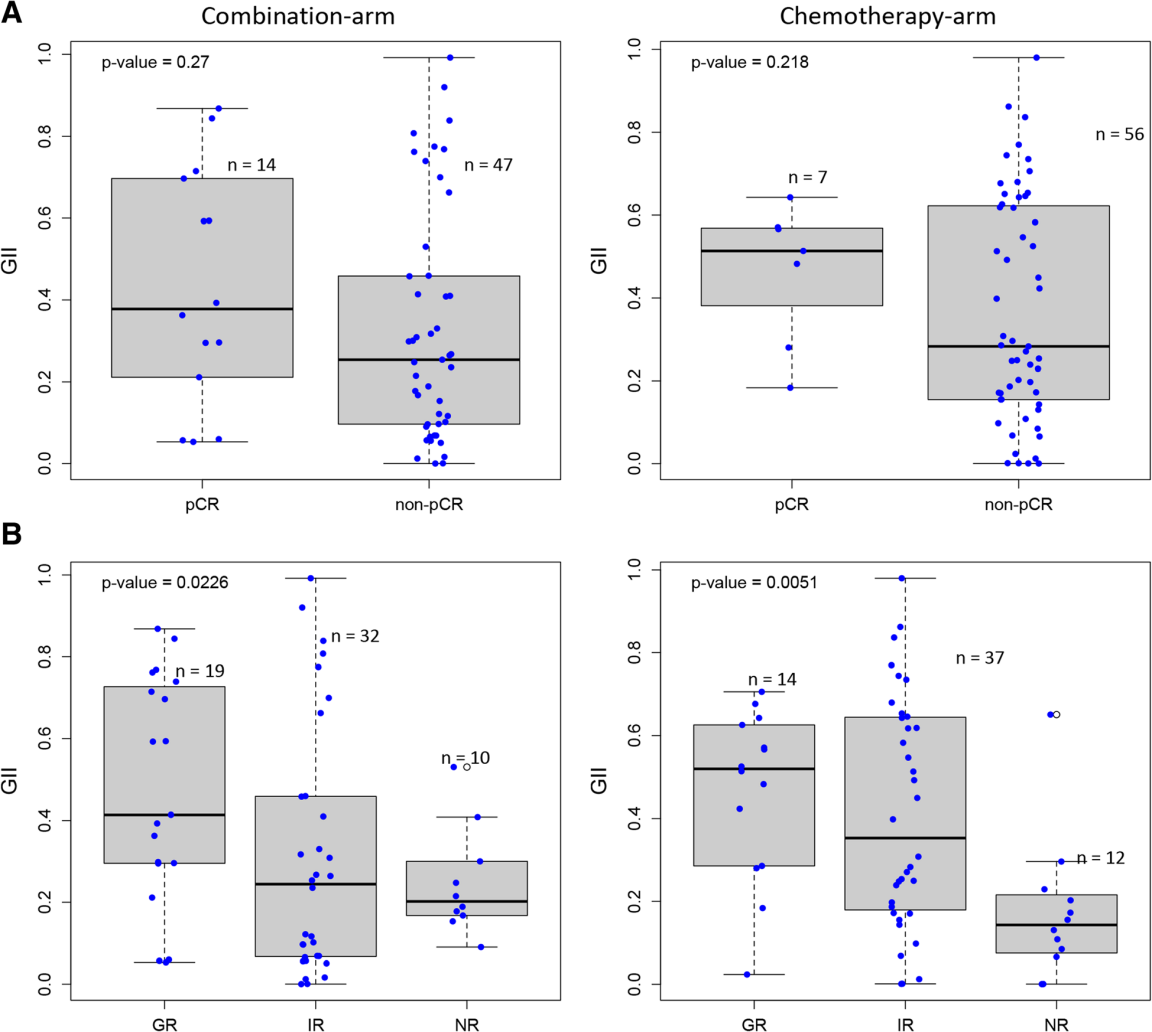
Degree of copy number aberrations between different response groups within each treatment arm. **a** Difference in genomic instability index (GII, *y*-axis) between patients obtaining pCR and non-pCR (*x*-axis). No significant difference was observed in either treatment arm (Student’s *t* test). **b** Significant difference in tumors’ GII between patients with good response (GR), intermediate response (IR), and no response (NR) (ANOVA test *p* value < 0.05) within both treatment arms

CNAs adjusted for tumor percent and average ploidy using the ASCAT algorithm were used to compute the GII as described in the “Methods” section. Since patients were randomized into treatment arms, the mean GII prior to treatment was similar in both arms (Additional file 1: Figure S1A). When comparing patients achieving pCR and non-pCR, at diagnosis, there was a small, but insignificant difference in mean GII between the two response groups (Fig. 1a, Student’s *t* test *p* value = 0.27 and 0.218 for the combination and chemotherapy arms, respectively). However, when the percentage shrinkage of the tumor was used to categorize the patients into GR, IR, and NR, there was a significant difference in mean GII between the three groups (Fig. 1b, ANOVA *p* value = 0.0226 and 0.0051 for the combination and chemotherapy arms, respectively). The GR tumors had a significantly higher GII than the IR and NR tumors, whereas the IR tumors had a greater spread of the GII at week 0. These observations were similar for both treatment arms.

**Fig. 2:**
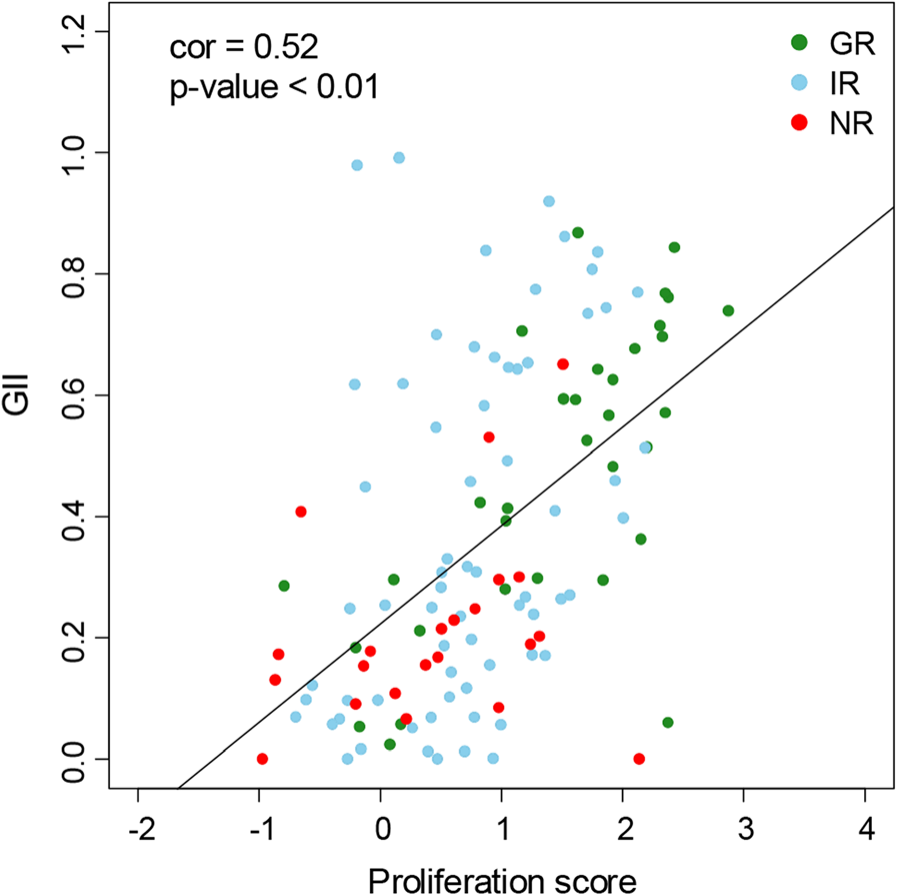
Genomic instability index (GII) as a function of proliferation score for with good response (GR, green), intermediate response (IR, light blue), and no response (NR, red) tumors for both treatment arms. Significant correlation was observed (Pearson correlation = 0.52, *p* value < 0.01)

The GII of untreated tumors was significantly correlated to the proliferation score obtained from mRNA expression (Fig. 2; Pearson correlation = 0.52, *p* value < 0.01). Thus, GR tumors have high GII and proliferation score, while NR tumors have low values of both scores. The IR tumors were more heterogeneous both regarding the GII and the proliferation score.

**Fig. 3:**
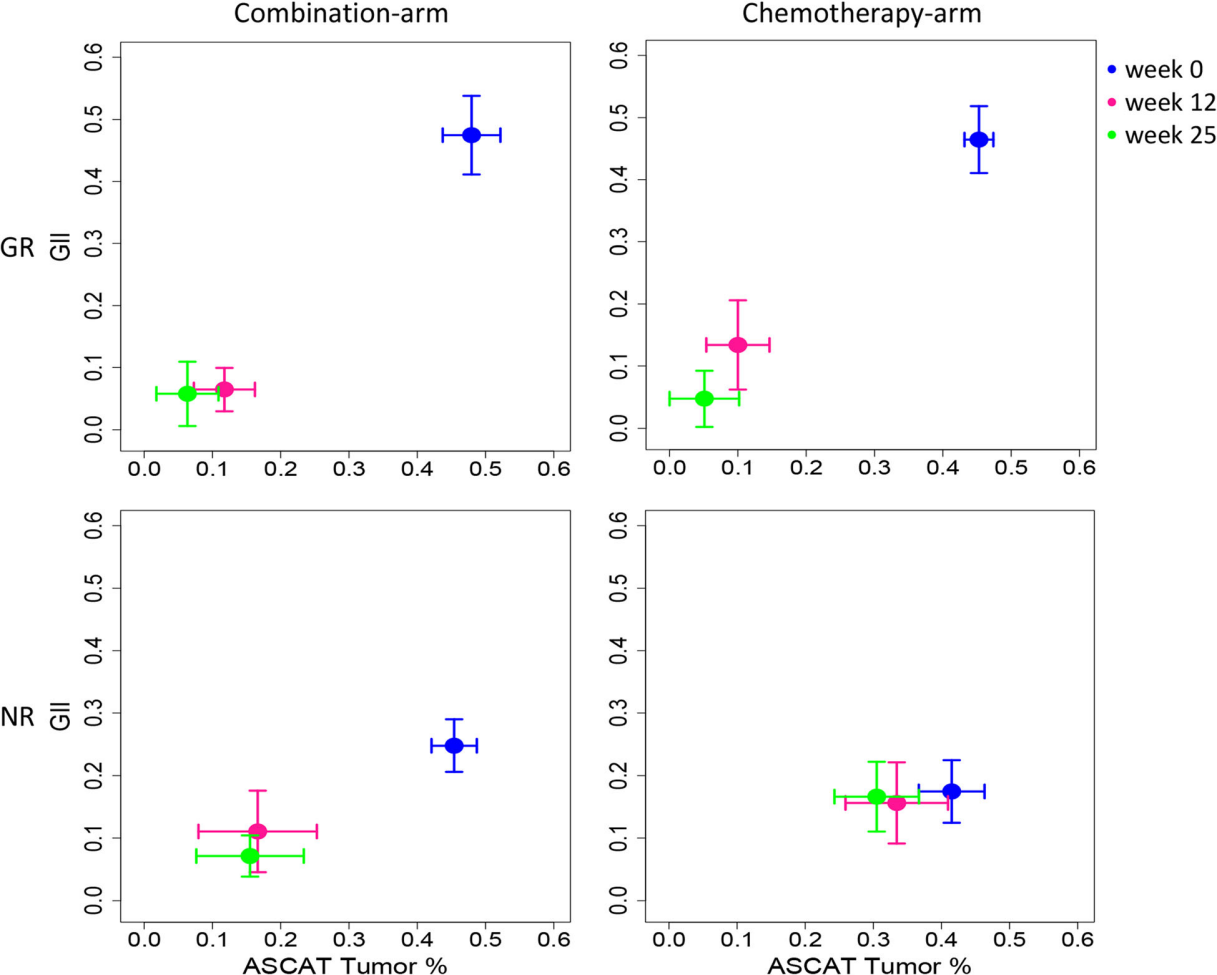
Mean genomic instability index (GII) versus tumor percentage (deduced from ASCAT) before, during, and after treatment, stratified on treatment arms. The top row shows that patients with good response (GR) independent of treatment arms have a higher mean GII, but similar average tumor percentage (bars indicating standard error), than patients with no response (NR) tumors (lower row) before any treatment (blue). After 12 weeks of treatment (pink), the mean GII and tumor percentage drastically gets reduced in the GR tumors (top row), and at the time of surgery (green), more or less all sign of tumor is lost in both treatment arms. Patients not responding to the combination therapy (bottom left plot) show a reduction in mean GII and tumor percentage after 12 weeks of treatment (pink), which halts until time of surgery (green). The bottom right plot reveals that the shift in mean GII and tumor percentage between the three time points is very low for NR tumors in the chemotherapy arm

When comparing the mean tumor percentage and mean GII before, during, and after treatment (Fig. 3), we observed differences between the two extreme responder groups, GR and NR. There was a significant difference in mean GII between the GR and NR tumors at week 0 (Student’s *t* test *p* value < 0.01). This was observed in both treatment arms. Already after the first cycle of treatment with FEC at week 12, GII and tumor percentage had decreased in GR tumors, for both treatment arms (Fig. 3). A more modest shift was seen for GR tumors after the second treatment regimen with taxanes at week 25. At time of surgery, tumors in the GR group had a tumor percentage and GII score close to zero. Compared to the GR tumors, the non-responders in the combination arm showed a more modest drop in GII and tumor percentage during the first cycle of treatment with FEC and no apparent drop during treatment with taxanes (Fig. 3). NR tumors in the chemotherapy arm showed small or no decrease in mean GII and tumor percent during both treatment regimens. In NR tumors, both aberrant tumor cells and genomic instabilities were still present at week 25 in both treatment arms.

Amplifications/gains and deletions were assigned an aberration score, + 1 for gain and − 1 for loss, respectively. The occurrence of alterations genome wide was calculated across all samples and plotted as frequency plots. Again, due to the randomization, similar aberration patterns across the whole genome were observed in the patients from both arms prior to treatment (Additional file 1: Figure S1B).

### Aberration patterns associated with responses to chemotherapy and combination treatment

**Fig. 4:**
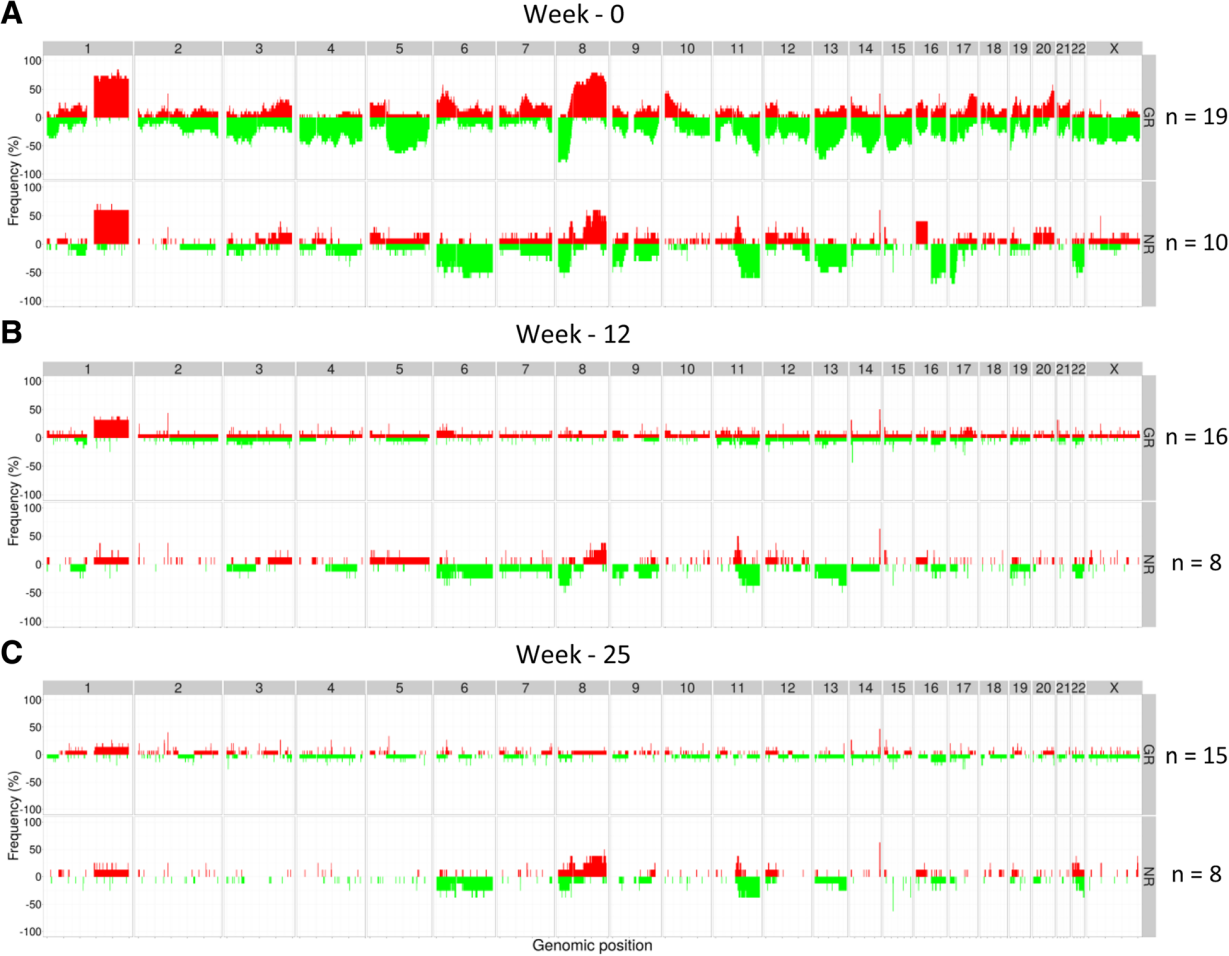
Frequency plots of genome-wide copy number aberrations (CNAs) in tumors at the time of diagnosis (**a**), after 12 weeks of treatment (**b**), and at the time of surgery (**c**) from patients in the combination arm. The *y*-axis indicates frequency (%) of tumors with gains (red) and deletions (green) sorted by genomic positions (*x*-axis) across all chromosomes (annotated at top of the plots). **a** Untreated tumors from good response (GR) tumors (*n* = 19, top plot) show a higher frequency of alterations genome wide, in comparison with no response (NR) (*n* = 10, bottom plot). Loci significantly associated to different responses are marked with asterisk. **b**, **c** Aberrations disappear during treatment for patients responding (top) to the therapy, while for the NR (bottom), several copy number changes are kept

We then focused our analyses separately on the two treatment arms. Breast carcinomas in the combination arm revealed different aberration patterns between the GR and NR, even before they were subjected to treatment. Prior to treatment, the genomes of good responders were more aberrant than those of non-responders (Fig. 4a). To identify genotypic alterations resulting in downstream phenotypic alterations, we filtered the copy number data based on correlation between logR estimates (tumor percent and ploidy adjusted) for each gene and its mRNA expression (Pearson correlation cutoff ≥ 50% correlation). The logR values of 109 genes, located at 25 unique genomic regions, were significantly different between the good responders and non-responders within the combination arm (Student’s *t* test, FDR *q* value < 0.05) (Fig. 4a, Additional file 2: Table S1). Deletions of chromosome 4q13.3, 10q26, 11p15, 12q13-q14, and 14q23-q31, as well as amplifications of 6p22-p12 and 20q13, were associated to good response.

Tumors with no response (NR) had less aberrant genomes prior to treatment (week 0), and fewer CNAs were associated with response ratio compared to GR tumors. No response was associated with deletions of 6p22-p12. Interestingly, in this locus, the tumor CN profiles of GR and NR exhibited an inverse aberration pattern. Amplification of 6p22-p12 was significantly associated with good response, and deletion was associated with no response to the combinatory therapy. These loci encompasses several interesting genes, including genes in the VEGF pathway such as *VEGFA*, *MAPK13*, and *MAPK14*, as well as genes in the major histocompatibility complex (MHC) I and II. *VEGFA* and *MAPK13* did not pass the mRNA expression correlation filter due to Pearson correlation below 50% (46% and 34%, respectively), but *MAPK14* did.

**Fig. 5:**
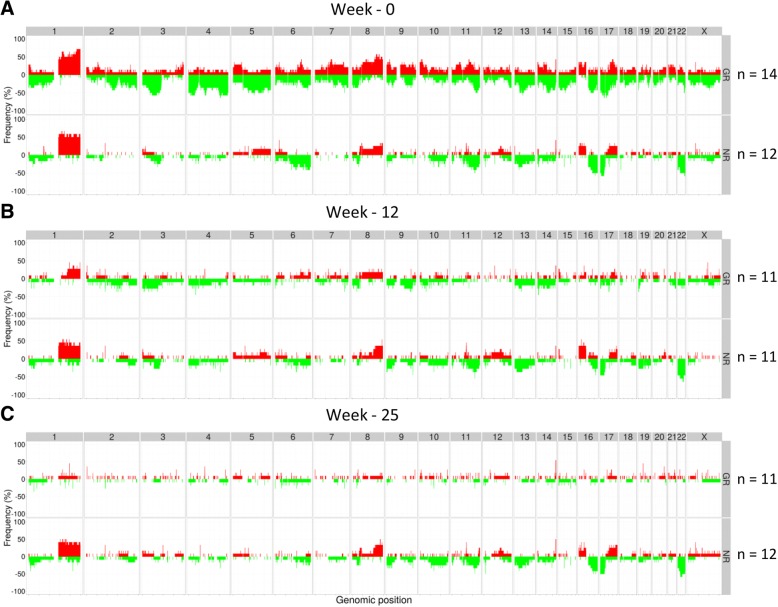
Frequency plots of genome-wide copy number aberrations (CNAs) in tumors at the time of diagnosis (**a**), after 12 weeks of treatment (**b**), and at the time of surgery (**c**) for patients treated with chemotherapy alone. The *y*-axis indicates frequency (%) of tumors with gains (red) and deletions (green) sorted by genomic positions (*x*-axis) across all chromosomes (annotated at top of the plots). Higher frequency of copy number changes is observed in untreated good response (GR) tumors (**a**, top) compared to no response (NR) tumors (**a**, bottom). During treatment (weeks 12 and 25), the GR tumors shrink and CNA frequency profiles lose their aberrations (**b**, **c**, top). Tumors not responding to treatment keep their aberrations during treatment (bottom)

Within the patient group treated with chemotherapy only, the frequency of CNAs was also different between the responding and non-responding tumors prior to treatment (Fig. 5a). Again, the responsive tumors showed a higher frequency of alterations genome wide compared to NR tumors. The association of CNAs in untreated tumors to the two extreme response groups was investigated, but no genes were significantly associated to response category after multiple testing correction (FDR *q* value < 0.05). Ninety-seven genes, located at 39 different loci, were found to differ significantly between the GR and NR (Student’s *t* test *p* value < 0.05) before multiple testing correction in the chemotherapy arm only (Additional file 3: Table S2).

### Treatment-specific alterations in aberration pattern

As expected, the good responders lose all aberrations during treatment and move towards a “normal” signal (Fig. 4b, c and Fig. 5b, c).

#### Combination arm

Certain alterations in the non-responding tumors disappear, while others seem to persist during treatment with chemotherapy in combination with bevacizumab (Fig. 4b, c). Aberration patterns in non-responding tumors within the combination arm at week 12 showed a frequency of gain > 30% at chromosomes 11q13.2 and 12p11.21 (details in Additional file 4: Table S3). Frequency of deletions > 30% were observed at chromosomes 6p21.33-p21.32, 8p, 11q13.5-q25, 13q31-q34, and 19q13. At the time of surgery, more than 30% of the NR tumors in the combination arm exhibited gain of chromosomes 8p11, 8q22-q23, 11q13.2-q13.3, and 22q12.1 and deletion of 6p12-p11, 6q21-q22, 6q24.2, 8p11.22, and 11q13.4-q25 (detailed overview Additional file 5: Table S4). Gain of 11q13.2 and deletion of 8p11.22, 11q14.1-q14.3, and 11q21-q25 were shared between NR tumors from week 12 and time of surgery (detailed overview Additional file 4: Table S3 and Additional file 5: Table S4). *CCND1*, *CTTN*, *FGF3*, *FGF19*, *ORAOV1*, *ANO1*, *FADD* gain, among others, were still present at week 25 in > 30% of the NR tumors in the combination arm.

#### Chemotherapy arm

After treatment with FEC, more than 30% of the non-responding tumors still exhibit gain of 1q, 8q23-q24, 16p13-p11, and 17q25.3 and deletion of e.g. 1q32.3, 6q16.2, 9p24.3, 9q33.2, 13q12-q14, 17p13.3-p11.1, and 22q (Additional file 6: Table S5). After treatment with taxanes, NR tumors had frequent (> 30%) deletions of e.g. 1p36, 1q32.3, 9p22.3, 9q33.2, 10q23.31, 13q, 16q, 17p, and 22q and gain of 1q, 4q12, 16p13.12, and 17q25 (Fig. 5b, c) (detailed overview in Additional file 7: Table S6). The specific aberrations mentioned above retained during and after treatment were unique to NR tumors treated only with chemotherapy.

#### Both treatment arms

Even though certain CNAs retained during treatment were specific to each treatment arm, some aberrations retained in the NR tumors after the first part of the treatment were common for both treatment arms. The common aberrations associated to poor response (NR) were deletion of 11q22.3-23.3 and 22q13.33 and gain of 1q23.2, 8q23-q24, and 11q13.3. The genes amplified in > 30% of NR tumors in both treatment arms at 11q13.3 included *ANO1* and its anti-sense product *ANO1-AS2*, *CTTN*, *FADD*, *FGF19*, *FGF3*, *LOC100127946*, and *PPFIA1* (detailed overview in Additional file 4: Table S3 and Additional file 5: Table S4).

### Clonal and subclonal tumor composition under treatment pressure

Estimating subclonal architecture in tumors during treatment is challenging due to decreasing tumor cellularity, as a consequence of response to treatment. However, by aggregating changes in copy number profiles across many samples (as described in detail in the “Methods” section), we identified genomic regions that are subject to copy number selection during treatment, resulting in the expansion of subclones bearing these CNAs. Similarly, we identified CNAs conferring sensitivity to treatment and, hence, preferential shrinkage of the subclones bearing these aberrations.

**Fig. 6:**
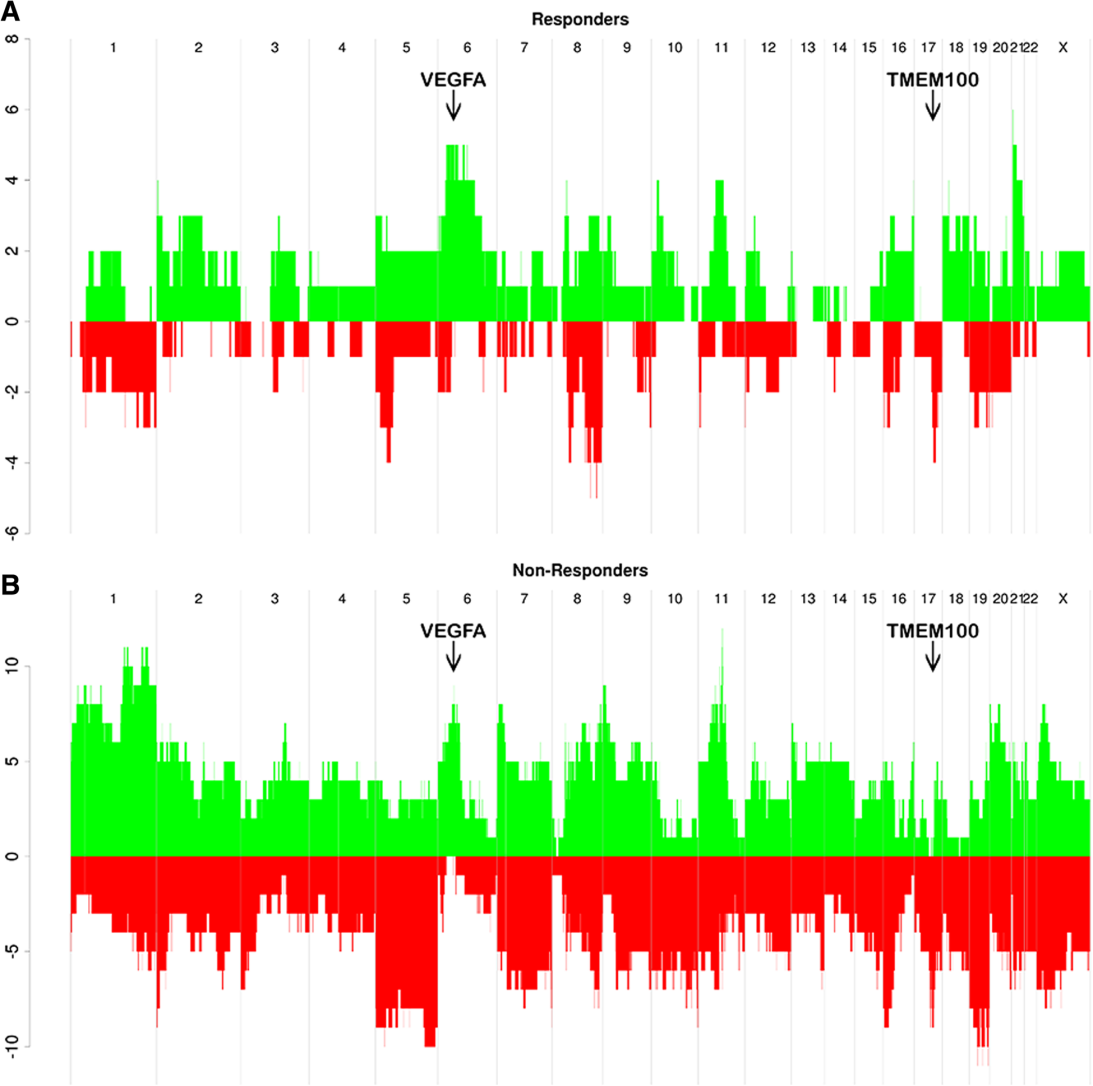
Number of patients showing an increase (green) or a decrease (red) in the subclonality of copy number gains genome wide between diagnosis and 12 weeks after treatment for responders (**a**) and non-responders (**b**). Significantly more patients showed an increase in the clonality of VEGFA gains and a decrease in the clonality of TMEM100 gains (arrows) across the whole cohort

We first estimated the aberrant cell fraction (ACF) of the reference sample at each time point 0 using the Battenberg output. Samples that are diploid in the reference sample (ploidy < 3) were used to identify segments that have just one aberrant copy number state, i.e., segments that are clonal and aberrant or that are a subclonal mixture of two copy number states and where one of the states is non-aberrant. Based on this, aberrant segments were categorized as clonal or subclonal and as either loss, gain, or LOH. For each segment, the fraction of cells bearing the CNA was estimated for each time point. The total number of samples that showed an increase or a decrease in clonality with time during treatment in each segment was calculated. Increase/decrease in subclonality was then determined separately in each 12- or 25-week sample, relative to the diagnosis sample. The number of increases/decreases was summed across all patients. We expect segments that have no selective pressure to have the same number of increases and decreases, on average, across all tumors. In this manner, we were able to identify subclonal events whose abundance changed with time. For instance, the subclonal fraction of cells containing copy number gains of 6p21.1, the locus which contains the VEGFA gene targeted by bevacizumab, were found to be increased at 12 weeks (FDR = 0.044, Fig. 6). Of 8 patients treated with combination therapy and with a gain at chr6:43 M (VEGFA), all showed an increase in subclonality at 12 weeks. Of 5 patients not treated with combination therapy and with a gain at chr6:43 M (VEGFA), all showed an increase in subclonality at 12 weeks. Neither of these gives FDR < 0.05 by themselves, but in combination, these 13 patients give an FDR of 0.044. The increase may be the result of either an increase in the proportion of cells bearing the amplification or the acquisition of further gains, leading to increased copy number. The increase was seen in both treatment arms. Of the 13 pre-treatment samples that had a gain at VEGFA, only 1 was a non-responder.

On the other hand, the frequency of17q21.32-q22 gains showed a significant decrease at 12 weeks (FDR = 0.037, Fig. 6), with the aberration peak occurring at *TMEM100*, an *ALK1* receptor signaling-dependent gene essential for vasculogenesis. This implies that cells bearing amplifications of *TMEM100* are particularly sensitive to the treatment regime. The decline of cells with gains of *TMEM100* is observed in patients in both the combination and the chemotherapy arms. The increase (green) or a decrease (red) in the clonality of copy number changes genome wide in responders and non-responders at each time point are given in Additional file 1: Figure S2.

## Discussion

Discovering molecular predictive markers, such as ER and HER2, with the corresponding therapies has, in addition to general improvement of chemotherapy regimens, improved long-term survival for breast cancer patients. Certain patients will still not respond to treatment or will acquire resistance. Tailored therapy may limit over-treatment of patients who can benefit from lower doses and less extensive treatment regimens. Achieving pCR after neoadjuvant therapy has been shown to improve patient prognosis, and markers such as Ki67 and breast cancer’s five molecular subtypes have been suggested as predictive for breast cancer patients [9, 10]. Furthermore, high-resolution molecular markers as the ones reported here are needed to improve prediction of response to various therapies, including antiangiogenic treatment. Breast cancer patients treated with neoadjuvant chemotherapy in combination with bevacizumab have shown improved pCR rates and our results are in line with other studies [15–18]. However, markers for selecting the appropriate patients for such therapy are missing.

Comparing CNA at DNA level in untreated tumors in the GR and NR groups of patients treated with bevacizumab revealed significant differences in genomic instability. GR tumors had high GII compared to NR tumors, independent of treatment arm. High GII is also significantly correlated with high proliferation, indicating that GR tumors are proliferative. Chemotherapy has been shown to have an increased efficacy on highly proliferative cells; thus, these tumors respond well to chemotherapy. Whether the high number of genomic alterations causes the increased proliferation or elevated proliferation leads to genomic disruption is unknown. Since more than half of the responding ER-positive tumors had a high GII and proliferation score, these parameters may have important effects on treatment response. This is in line with the fact that the most prominent changes in gene expression were found in Luminal B tumors [25], which are often ER-positive tumors with a high GII and proliferation score. Not surprisingly, during treatment, GR tumors move towards a more normal cellular state with tumor cell percentage and a GII equal to zero. NR tumors are more likely to retain tumor cells and aberrations during treatment and have a smaller shift in tumor percentage and GII in both treatment arms. Studying copy number aberration patterns revealed amplifications and/or deletions of genes that were significantly associated with response. Few amplified/deleted genes overlapped between the two treatment arms, and mean logR values were not significantly different between the three response groups within the chemotherapy arm.

For tumors treated with chemotherapy in combination with bevacizumab, multiple loci were found to differ significantly in copy number state between GR and NR. Amplification of *MAPK14* was associated with improved response, while deletion of the same gene was associated with lack of response. The MAPK14 protein (p38) is a downstream target of VEGF. It is thought that MAPK14 and VEGF are in a regulatory circuit, whereby inhibition of MAPK14 enhances VEGF-induced angiogenesis and decreases vascular permeability [11]. Studies have shown that increased vascular permeability leads to leaky vessels that can increase accumulation of the therapeutics in the tumor (enhanced permeability and retention (EPR) effect). Yanagisawa and colleagues showed that treatment of human breast cancer xenografts with paclitaxel in combination with bevacizumab increased tumor concentration of paclitaxel compared to treatment with paclitaxel alone. Bevacizumab decreased vascular permeability, thus inhibiting the efflux of paclitaxel leading to an increased efficacy [26]. Interestingly, GR and NR tumors in the combination arm also showed inverse aberration patterns at chromosome 6p22-p12. There were no CNAs found significantly associated with response in the chemotherapy arm after multiple testing correction.

GR tumors in both treatment arms showed a reduction in aberrations during treatment, while NR tumors retained aberrations at several loci after 12 weeks of treatment, as well as at the time of surgery. Within the combination arm, focal amplification of 11q13.3 was kept at a high frequency (> 30%) in the non-responding tumors during treatment. Curtis et al. hypothesized that the 11q13/14 amplicon may be driven by a cassette of genes, rather than one driver oncogene [27]. Aberrations retained during treatment could be markers for patients who could benefit from a different treatment regimen, including inhibitors of Cyclin D1 activity, such as palbociclib, an inhibitor of both CDK4 and 6, recently approved by FDA for treatment of patients with advanced breast cancer in combination with antihormonal therapies.

Most tumors are composed of several subclonal populations. Some of these populations will have stronger resistance to the applied treatment regime and will therefore constitute an increasing proportion of the tumor mass as treatment progresses, while sensitive populations will decline. Here, we identified genomic regions that are subject to selection leading to the expansion of subclones bearing CNAs in the course of the treatment. Changes were observed in the subclonal composition of tumors across both treatment arms. Cells bearing gains of 17q21.32-q22, contains among other *TMEM100*, were particularly sensitive to treatment. On the other hand, a net increase in the proportion of cells bearing gains of 6p21.1, harboring the *VEGFA* gene, was observed across tumors (Fig. 6). It is consistent with the previous finding that patients with amplified *VEGFA* have worse progression-free and overall survival under treatment with paclitaxel and bevacizumab [28]. It appears that, while gains at this locus are associated with good response during the period of this study, those cells bearing the aberration are more likely to survive treatment and may therefore, perversely, result in worse outcome.

## Conclusions

This study confirms previous observations that highly proliferative tumors may exhibit immediate response to chemotherapy during treatment, which follows the known mechanisms of action of chemotherapeutic agents. In this study as well, the largest decrease in tumor volume was observed after the first treatment cycle with FEC, which is cytostatic by nature. Interestingly, the gross number of CNAs presented in the form of GII correlates with proliferation, suggesting that increased number of cell divisions may lead to the propagation of CNAs, and/or certain CNAs may be selected to drive proliferation further. While these unspecific effects were observed in both treatment arms (stronger in responders and to a lower degree in non-responders) and may be attributable to the effect of chemotherapy, in the combination arm, we observed several CNAs specifically associated to response already prior to treatment. Amplification of *MAPK14* was associated with improved response, while deletion of the same genes was associated with lack of response. *MAPK14* encodes the p38 protein, a downstream target of VEGF. VEGF and p38 have been shown to be involved in vascular permeability, cell motility, and regulation of cell junctions. Furthermore, some of these CNAs, like gains of 6p21.1, which contains the VEGFA gene, were in this study seen as subjected to subclonal expansion. Others, like gains of 17q21.32-q22, harboring the *TMEM100* gene, were residing in subclones disappearing in the course of the treatment. Taken together, these results highlight the importance of dissecting the tumor heterogeneity and molecular profile to assign the best-fitted treatment to each patient. Even though sequential biopsies are invasive procedures, it could have major impact on treatment response prediction and prognosis of outcome.

## Additional files

Additional file 1: **Figure S1.** A. GII in untreated tumors within the two therapy arms. B. Frequency plots of genome wide aberration score for tumors at the time of diagnosis in the Combination arm (top) and the Chemotherapy arm (bottom). **Figure S2.** Number of patients showing an increase (green) or a decrease (red) in the clonality of copy number changes genome wide between diagnosis and 12 weeks after treatment: copy number losses in responders (A) and non-responders (B); LOH in responders (C) and non-responders (D); gains in responders (E) and non-responders (F). (PDF 725 kb)

Additional file 2: **Table S1.** Genes and loci with significantly difference in logR estimates between the two extreme response groups GR and NR (t-test, FDR *p*-value < 0.05), unique for the Combination arm. (XLSX 11 kb)

Additional file 3: **Table S2.** Genes and loci with significantly difference in logR estimates between the two extreme response groups GR and NR (t-test, FDR p-value < 0.05), unique for the Chemotherapy arm. (XLSX 13 kb)

Additional file 4: **Table S3.** Aberrant genes in > 30% of the samples at week 12 within the Combination arm. (XLSX 26 kb)

Additional file 5: **Table S4.** Aberrant genes in > 30% of the samples at week 25 within the Combination arm. (XLSX 25 kb)

Additional file 6: **Table S5.** Aberrant genes in > 30% of the samples at week 12 within the Chemotherapy arm. (XLSX 71 kb)

Additional file 7: **Table S6.** Aberrant genes in > 30% of the samples at week 25 within the Chemotherapy arm. (XLSX 72 kb)

## Abbreviations

ACF: Aberrant cell fraction
ANOVA: Analysis of variance
ASCAT: Allele-specific copy number analysis of tumors
ASPCF: Allele-specific piecewise constant fitting
CNA: Copy number alteration
EPR: Enhanced permeability and retention
GII: Genomic instability index
GR: Good response
IR: Intermediate response
NR: No response
pCR: Pathological complete response
VEGFA: Vascular endothelial growth factor A
VEGFR: VEGF receptor
