# Supplementary material for: Time series analysis of neoadjuvant chemotherapy and bevacizumab-treated breast carcinomas reveals a systemic shift in genomic aberrations
Source: Genome Med. 2018 Nov 29;10:92. doi: 10.1186/s13073-018-0601-y (PMC6262977; doi:10.1186/s13073-018-0601-y)
Supplement: Supplementary file 1 — Figure S1. A. GII in untreated tumors within the two therapy arms. B. Frequency plots of genome wide aberration score for tumors at the time of diagnosis in the Combination arm (top) and the Chemotherapy arm (bottom). Figure S2. Number of patients showing an increase (green) or a decrease (red) in the clonality of copy number changes genome wide between diagnosis and 12 weeks after treatment: copy number losses in responders (A) and non-responders (B); LOH in responders (C) and non-responders (D); gains in responders (E) and non-responders (F). (PDF 725 kb) [file 13073_2018_601_MOESM1_ESM.pdf]

Additional file 1: Figures S1 and S2

Time series analysis of neoadjuvant chemotherapy and bevacizumab treated breast carcinomas reveals a systemic shift in genomic aberrations

Elen Kristine Höglander[1,2], Silje Nord[1,2], David C Wedge[3], Ole Christian Lingjærde[2,4], Laxmi Silwal-Pandit[1,2], Hedda vdL Gythfeldt[1,5], Hans Kristian Moen Vollan[1,2,5], Thomas Fleischer[1,2], Marit Krohn[1,2], Ellen Schlitchting[6], Elin Borgen[7], Øystein Garred[7], Marit M Holmen[8], Erik Wist[5], Bjørn Naume[2,5], Peter Van Loo[9], Anne-Lise Børresen-Dale[1,2], Olav Engebraaten[2,5,], Vessela Kristensen[1,2,10,*]*

[1] Department of Genetics, Oslo University Hospital, Oslo, Norway.
[2] KG Jebsen Center for Breast Cancer Research, Institute for Clinical Medicine, University of Oslo, Norway.
[3] Big Data Institute, University of Oxford, Oxford, UK.
[4] University of Oslo, Biomedical Informatics, Department of Informatics and Centre for Cancer Biomedicine, Oslo, Norway.
[5] Department of Oncology, Oslo University Hospital, Oslo, Norway.
[6] Section for Breast and Endocrine Surgery, Oslo University Hospital, Oslo, Norway.
[7] Department of Pathology, Oslo University Hospital, Oslo, Norway.
[8] Department of Radiology, Oslo University Hospital, Oslo, Norway.
[9] Cancer Research UK London Research Institute, London, UK.
[10] Department of Clinical Molecular Biology (EpiGen), Divison of Medicine, Akershus University Hospital, Lørenskog, and University of Oslo, Norway.

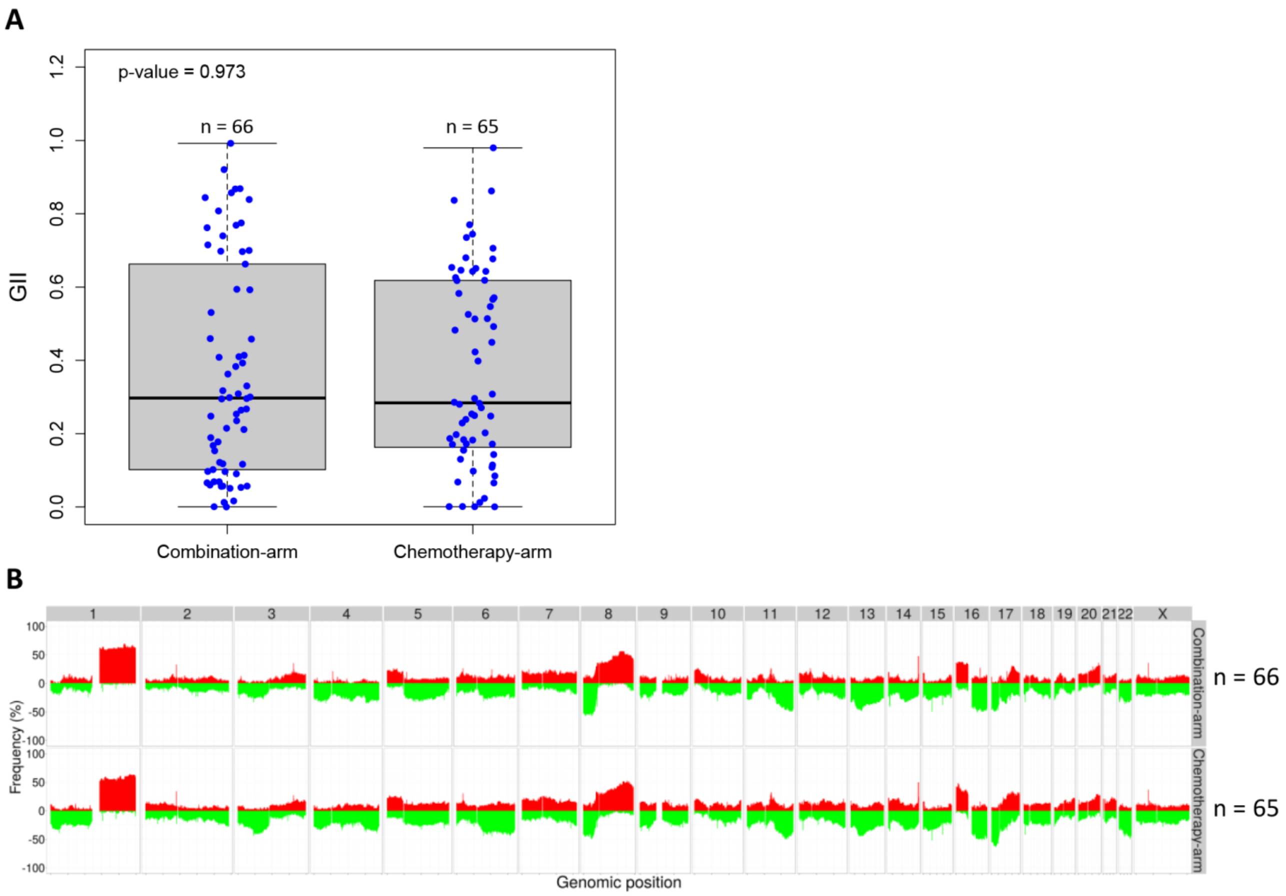

Figure S1: A. GII in untreated tumors within the two therapy arms. **B.** Frequency plots of genome wide aberration score for tumors at the time of diagnosis in the Combination-arm (top) and the Chemotherapy-arm (bottom).

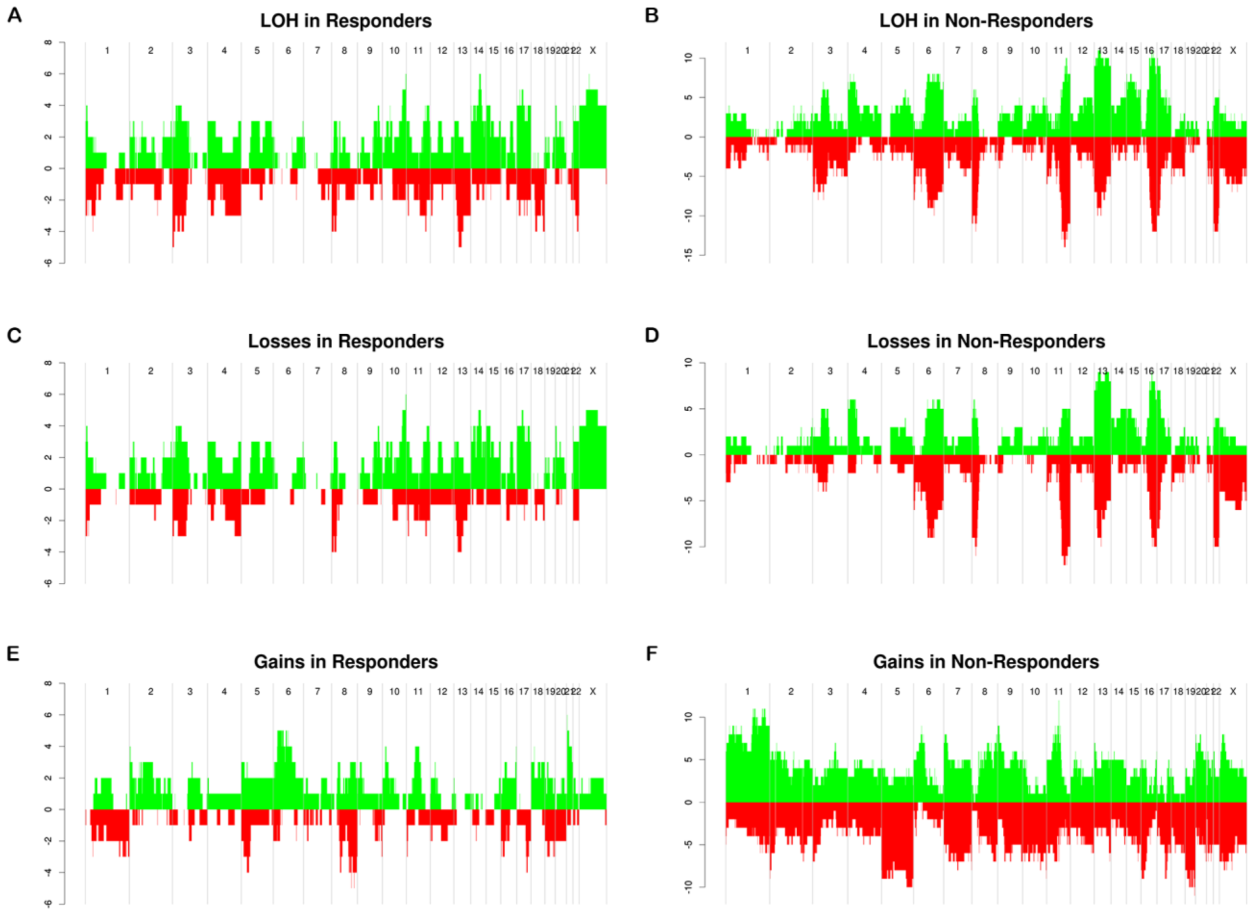

Figure S2: Number of patients showing an increase (green) or a decrease (red) in the clonality of copy number changes genome wide between diagnosis and 12 weeks after treatment: copy number losses in responders (**A**) and non-responders (**B**); LOH in responders (**C**) and non-responders (**D**); gains in responders (**E**) and non-responders (**F**).
